# Trap Count Characteristics of the Flat Grain Beetle *Cryptolestes pusillus* in Bulk Paddy Grain: Relationships with Insect Density

**DOI:** 10.3390/insects16070730

**Published:** 2025-07-17

**Authors:** Zhongming Wang, Miao Cui, Jiangtao Li, Huiling Zhou, Zhengyan Wang

**Affiliations:** 1 National Engineering Research Center of Grain Storage and Logistics, Academy of National Food and Strategic Reserves Administration, Beijing 100037, China; wzm@ags.ac.cn (Z.W.); cm@ags.ac.cn (M.C.); 2 Institute of Grain Storage and Logistics, Beijing 100037, China; 3 School of Artificial Intelligence, Beijing University of Posts and Telecommunications, Beijing 100876, China; 4 School of Intelligent Engineering and Automation, Beijing University of Posts and Telecommunications, Beijing 100876, China; huiling@bupt.edu.cn; 5 School of Food Science and Technology, Henan University of Technology, Zhengzhou 450001, China; zywang@haut.edu.cn

**Keywords:** stored-product pest, *Cryptolestes pusillus*, trap count, insect detection, spatial distribution, insect density

## Abstract

We studied the characteristics of adult flat grain beetles *Cryptolestes pusillus* Schönherr in trap counts and their relationship with insect densities using electronic probe traps in experimental bins, which contained approximately 1.1 tons of paddy (Changlixiang) with moisture contents of 10.7% and 14.0% and insect densities of 0.1, 1.0, and 5.0 adults/kg. Inside each bin, we vertically installed three layers of electronic probe traps. We installed five traps at the center and half-radius of each layer of the bin. We undertook measurements of daily trap counts, grain temperature, and intergranular relative humidity at each trap location for up to 10 days, and 1.0 kg of paddy was collected from each trap location. At each of the introduced insect densities, we detected beetles using electronic probe traps. When insect density was 0.1 adults/kg, we could not detect the existence of insects in 1.0 kg samples. It was found that the trap counts were spatially nonuniformly distributed, and there was a weak correlation between different locations. There was a regularity in the temporal distribution of trap counts, which was significantly influenced by the paddy moisture content. Except for the insect density, the moisture content, grain temperature, and intergranular relative humidity at the trap location significantly affected trap counts. The distribution pattern of beetles in paddy bulks was found and aggregated by analyzing trap counts. There was a correlation between trap counts and insect densities in grain bulks, but this correlation varied significantly across different locations.

## Simple Summary

This work identifies issues regarding the practical assessment of stored-product pest occurrence measures based on trap counts. Flat grain beetle, *Cryptolestes pusillus* Schönherr, is a common and serious stored-product insect pest. We characterized the distribution of *C. pusillus* adults in paddy bulks (Changlixiang) under different insect densities and grain moisture content levels using electronic probe traps and investigated the relationship between trap counts and insect densities under different storage conditions. It was discovered that trap counts were not stable during the whole detection period and were influenced significantly by grain moisture content. It was inaccurate to infer distribution dynamics only from trap counts over a specific period. Estimates of insect occurrence based on trap counts collected by only one electronic probe trap were inaccurate because of the aggregate distribution of insects, however, greater numbers of traps spaced at different locations increased accuracy. Results provide a theoretical basis for insect occurrence assessment models based on trap counts in bulk grain.

## 1. Introduction

The distribution pattern of insect pests within bulk stored grain is a critical ecological characteristic of each species [1,2]. Understanding distribution patterns is crucial when designing effective insect-monitoring programs and enabling more effective monitoring of pest behavioral patterns during grain storage. Distribution frequency and aggregation distribution patterns are assessed using sampling. Characteristics of adult insect distribution has been investigated under laboratory tests, which have indicated that those pests commonly exhibit aggregated spatial distributions within grain bulks, preferring middle and upper layers over bottom layers, and that this aggregation is strong at low densities and weakens as density is increased [3,4,5,6,7,8].

Electronic probe traps are a sensitive and efficient automatic detection device for insects inside grain storage facilities. Refs. [7,9,10] studied the spatial distribution pattern of adult rusty grain beetles in bulk wheat using electronic probe traps and manual sampling with a 15 kg sample. They showed that both methods yielded similar results when determining spatial distribution patterns of the insects. However, the question of how to estimate an actual population density within a grain mass using trap counts has always been challenging, due to the complex relationship between trap counts, pest activity and population density [8,10]. Therefore, determining the characteristics of trap counts from electronic probe traps is necessary when designing trap-count-based insect occurrence assessment programs for bulk stored grain. Few published studies have investigated the distribution of trap counts at different beetle densities and grain moisture content levels [8].

Flat grain beetle, *Cryptolestes pusillus,* is one of the most common and serious stored-product insect pests in China, infesting various agricultural products, including grains, oilseeds, beans, and dried fruits, as well as their processed products, sometimes in extremely high population densities [11]. In addition to direct consumption, these insects cause heat points and fungal infections in stored grain. To mitigate effects on stored grain, managers employ fumigation as a prevention and control measure [12]. Because of insect resistance to phosphine fumigant, monitoring has become a crucial aspect of managing insects in stored grain [13]. Few studies, however, have focused on the distribution of adult flat grain beetles in grain. Studies have primarily explored the occurrence and distribution patterns of common stored-product pests in stored wheat, while few have examined the spatial distribution characteristics of insects in paddy (Changlixiang), which is one of the staples of the human diet [14].

The objectives of this study were to (1) characterize the trap counts of adult flat grain beetles in paddy bulks under different insect densities and grain MC levels using electronic probe traps, (2) to analyze the factors that affect the trap counts, and (3) to investigate the relationship between trap counts and insect densities under different storage conditions in the laboratory.

## 2. Materials and Methods

### 2.1. Insects

We selected 2-week-old adults of *C. pusillus* from the laboratory of the Academy of National Food and Strategic Reserves Administration, China. The rearing conditions were 25 ± 3 °C and 65 ± 5% RH, and the diet consisted of whole wheat flour, oatmeal, wheat grain, and yeast in a ratio of 46:40:1:5. We used a gentle vacuum to collect the beetles and placed them in four glass bottles covered with mesh. Each bottle contained 0.5 kg of the same paddy used in this study. We placed the bottles in the bins to ensure that the selected beetles were acclimated to the testing conditions for 24 h.

### 2.2. Bins and Paddy

**Figure 1:**
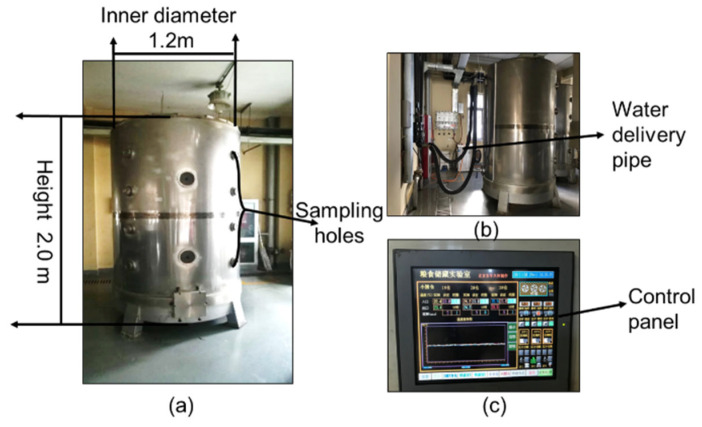
Experimental bins. (**a**) The picture of the bin; (**b**) The picture of water delivery pipe; (**c**) The picture of control panel.

Data of trap counts, grain temperature, and intergranular relative humidity were collected from three identical metal bins with a height of 2 m and an inner diameter of 1.2 m (Figure 1). The bins had double walls with a 10 cm gap between them, which was filled with water to the top; the water temperature was maintained at 25 ± 3 °C to regulate the paddy temperature. The bins were loosely covered with lids during the experiment to prevent insects from escaping while allowing for air exchange. Samples were collected manually through the sampling holes from all four layers along the walls. The four sampling holes were distributed evenly in each layer across the four radial directions. The distance between the two sampling holes in the vertical direction was 0.4 m. This was used to maintain the grain temperature.

The paddy (Changlixiang) was sampled from Hebei Province, China. Two MC levels were used in this experiment, 10.7% and 14.0%. Before the paddy was used, it was sterilized with phosphine fumigant and then warmed to 25 °C. To adjust its MC levels, a polyvinyl chloride (PVC) film was spread over the fumigated paddy on the laboratory floor and the paddy was sprayed with the specified amount of distilled water (approximately 40 kg water per 1000 kg paddy). The paddy was periodically raked manually for 10 days. The paddy was sealed in a plastic bag and stored in the laboratory for 1 month before it was loaded into bins.

### 2.3. Electronic Probe Traps and Insect Sampling

**Figure 2:**
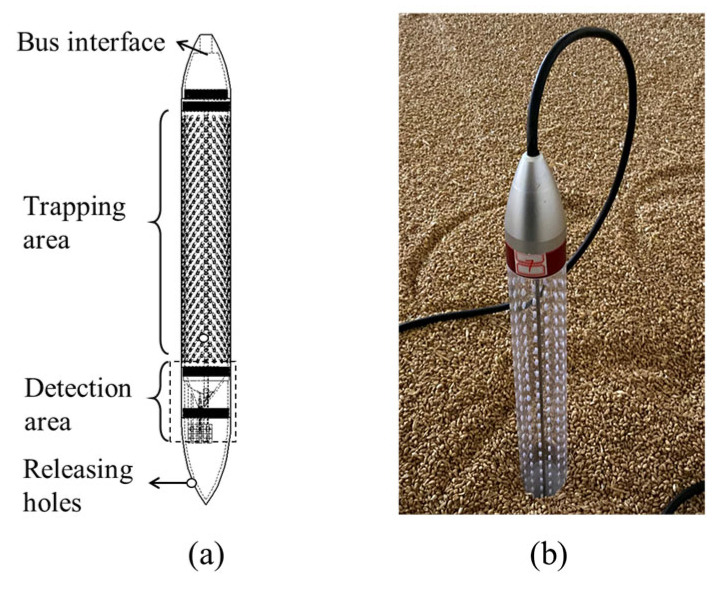
(**a**) The structure of the electronic trap used in this study; (**b**) The picture of the trap deployed in a grain storage warehouse.

**Figure 3:**
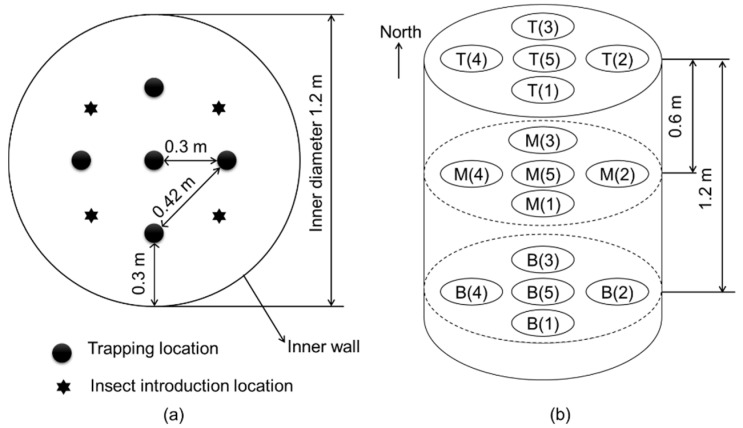
(**a**) Locations of insect introduction and electronic probe traps. (**b**) The deployment of traps. T, M, and B are the top, middle, and bottom layers, respectively. The numbers inside brackets are the order of trap locations.

Electronic probe traps [15], as shown in Figure 2, were used to count trapped beetles, simultaneously recording the signals triggered by the captured beetles and the temperature and humidity in the grain mass at the trap location. The trap could record the signals triggered by the captured adults when they fell between the detection area. To release the captured beetles, holes were made at the bottom of the traps (Figure 2a). Data were uploaded three times per hour. Figure 3 illustrates the trap deployment scheme. The top, middle, and bottom layers of the paddy bulk had 15 evenly placed traps. Each layer had one trap at the center and four traps at the half-radius in all four directions.

We used a grain probe to collect the samples using a gentle vacuum through sampling holes in the bins (Figure 1) and the sampling unit was 1 kg [16]. Beetles in each sample were collected and counted with the help of a sieve with apertures of 2.5 mm. The sample including beetles was then returned to the paddy bulk from the top of the bin. To reduce the effect of manual sampling on the distribution status of beetles in bins, we took manual sampling on day 10 after insect introduction.

### 2.4. Test Procedure

The experiment was conducted from late autumn (November 2021) to spring (April 2022). Each bin was loaded with approximately 1100 kg of paddy to a height of 1.8 m. During the process of loading paddy, we set 15 traps. We leveled the grain surface and stabilized the grain temperature at 25 ± 3 °C. The economic threshold for prevention and control of *C. pusillus* is five individuals per kg [16]. Therefore, we tested insect densities of 0.1, 1.0, and 5.0 adults/kg of paddy. The first group of 110 beetles was released at four positions on the surface of the grain (Figure 3a). Each bin had an insect density of 0.1 adults/kg. Ten days after the initial insect introduction, we sampled grains from 15 trap locations (Figure 3b). Then, we introduced the second group of 990 beetles to the same positions 24 h after sampling, and the insect density was 1.0 adults/kg. The procedure was repeated. Finally, a third group of 4400 adults was introduced to the bulk to create an insect density of 5.0 adults/kg. The procedure was repeated.

We conducted two MC and three beetle density experiments. The experiments with paddy with 10.7% MC had three replicates. The experiments with paddy with 14.0% MC had two replicates.

### 2.5. Data Analysis

We evaluated detection success of the electronic probe trap by calculating the average number of traps that detected beetles each day and the average total daily trap counts in each bin. We evaluated detection success of manual sampling by counting the number of samples in which beetles were detected and the number of detected beetles in each bin. For each replicate, we took 15 samples according to the manual sampling method. We drew the curves of the total daily trap counts for each replicate under different insect densities to demonstrate the time distribution characteristics of trap counts.

To determine where the beetles had been captured inside the bin, we calculated the percentage of capture at different layers (CP_L_) for each replicate, as follows:

(1) $${\mathrm{CP}}_{L}=\frac{\mathrm{CL}}{T}\;\times \;100,$$

where CP_L_ is the capture percentage at the specified layer (%), CL is the total trap counts in the 10-day trapping period from all of the traps in the specified layer L, and T is the total trap counts from all of the traps in the bin. For each replicate, we individually calculated the CP_L_ at the top, middle, and bottom layers.

Violin plots were drawn for the daily trap counts from each trap deployed in the top, middle, and bottom layers to analyze the spatial distribution of daily trap counts. The geostatistical method of Jian et al. (2011) [3] was used. The correlation coefficient for the following pairs of daily trap counts was calculated as follows: [H(0), H(30)], [H(0), H(42)], [V(T), V(M)], [V(M), V(B)], [V(T), V(B)], [V(0), V(60)], and [V(0), V(120)]. H is the number of daily trap counts in the horizontal direction, and V is the number of daily trap counts in the vertical direction at the trap location. The first bracket denotes the number of the start location, and the second bracket specifies the distance between the start trap and the second trap location in the indicated direction [3]. For instance, [H(0), H(30)] indicates that data at location H(0) were joined with data 30 cm away in the horizontal direction. A 30 cm distance horizontal direction existed between the center and the half-radius. In this pair, H(0) indicated the trap count for that day at the center. A 42 cm distance in the horizontal direction existed between the two adjacent half-radii locations (Figure 2). The top, middle, and bottom layers are indicated in the brackets by T, M, and B, respectively. For instance, [V(M), V(B)] compares the middle and bottom layer trap counts for the day in the vertical direction.

The significance of factors affecting trap counts was analyzed. A factorial test to analyze the effect of the following factors on daily trap counts was undertaken: insect density, grain temperature, MC, and humidity. Grain temperatures were grouped according to temperature intervals of 0–20 °C, 20–25 °C, 25–30 °C, and 30–40 °C, and according to humidity intervals of 0–50%, 50–60%, 60–70%, and 70–80% to examine whether the grain temperature and humidity had a significant effect on trap counts. Heat maps of the Pearson correlation coefficient between daily trap counts, grain temperature, and humidity obtained from each trap under different insect densities further illustrated correlations.

To characterize the aggregation distribution pattern of *C. pusillus* adults in paddy bulks, we calculated Lloyd’s index of mean crowding [17] and Taylor’s power law [18] using trap counts. Trap count data from the 15 traps in each bin were used as the sample set for the calculation on sampling days. For each sample set, Lloyd’s index of mean crowding was calculated as follows:

(2) $$I_{L}=\overset{-}{\;x}+\left(\frac{s^{2}}{\overset{-}{x}}\right)\;-\;1,$$

where I_L_ is Lloyd’s index of mean crowding, $\overset{-}{x}$ is the mean of the trap counts of the sample set, and s^2^ is the variance. Then, we adopted the Iwao linear regression method [19] to determine the index of basic contagion (b_0_) (i.e., mean crowding of individuals) and the density–contagiousness coefficients (b_1_), i.e., patchiness of clusters, from the regression models:

(3) $$I_{L}=b_{0}+b_{1}\overset{-}{x},$$

where b_0_ denotes the size of the individual clumps that make up the basic unit of the spatial pattern. A value of b_0_ = 0 indicates that an individual is the basic component, b_0_ > 0 indicates that a colony is the basic component, and b_0_ < 0 indicates the tendency for individual repulsion. A value of b_1_ > 1 denotes a series of aggregated distributions, a value of b_1_ = 1 denotes random distribution, and a value of b_1_ < 1 denotes uniform distribution.

Taylor’s power law expressed in the logarithmic form [3,8] was used to fit the data, as follows:

(4) $$\ln s^{2}=\ln a+b\;\ln\;\overset{-}{x},$$

where lna is the intercept and b is the slope or specific aggregation index. We used the value of b to categorize uniform, random, and aggregated spatial patterns of insect distribution based on b being <1, =1, and >1, respectively [20].

The Pearson correlation coefficient was associated with insect densities. Frequencies of trap counts were calculated to find the relationship between insect densities and the frequencies (TF_D_) at each location under different MC levels, as follows:

(5) $$TF_{D}=\frac{\mathrm{Total}\;\mathrm{of}\;\mathrm{trap}\;\mathrm{counts}\;\mathrm{in}\;\mathrm{the}\;\mathrm{trappling}\;\mathrm{period}\;\mathrm{of}\;D\;\mathrm{days}}{\mathrm{Trapping}\;\mathrm{period}\;\mathrm{of}\;D\;\mathrm{days}}$$

where TF_D_ is the average value of trap counts in the specified trapping period (A/day). We tested the trapping periods in the past 3, 5, 7, or 10 days to find the highest relationship between the insect densities and frequencies of trap counts. We also calculated the average values of the Pearson correlation coefficient associated with the introduced insect density and the TF_D_ at each location in the past 3, 5, 7, and 10 d. Then, we calculated the Pearson correlation coefficient between the TF_D_ with the highest coefficient and the insect density at each location.

A paired *t*-test was adopted for the following data analysis. Data were analyzed with custom Python scripts (Version 3.5.2), primarily using Statsmodels (https://github.com/statsmodels/statsmodels/, Version 0.13.3, accessed on 2 November 2022), Scipy (https://scipy.org/, Version 1.8.0, accessed on 4 February 2022), Pandas (https://pandas.pydata.org/, Version1.5.0, accessed on 3 October 2022), and Seaborn (http://seaborn.pydata.org/, Version 0.12.0, accessed on 19 September 2022) packages.

## 3. Results and Discussion

### 3.1. Sensitivity of Two Detection Methods

At all insect densities, electronic probe traps detected beetles (Table 1). The average of total daily trap counts increased with the increase in insect density; however, we observed significant differences in the total daily trap counts at the same insect density with different MC levels (paired *t*-test, *t* = −10.690, *p* < 0.001). When the insect density was 0.1 adults/kg, no beetles were detected in the 75 samples taken manually. When the density was raised to 1.0 adults/kg, beetles were detected in only 5 of 75 samples by manual sampling. When the density was 5.0 adults/kg, beetles were detected in 36 of 75 samples, and the estimated densities of the 15 samples were all lower than the introduced densities. Deploying multiple traps in grain bulks for insect detection was more sensitive than manual sampling with a unit of 1.0 kg paddy; electronic probe trapping was more effective than manual sampling at insect densities less than 5.0 adults/kg. These results are consistent with the conclusions of Jian et al. [7].

### 3.2. Temporal Distribution of Adults Characterized by Trap Counts

**Figure 4:**
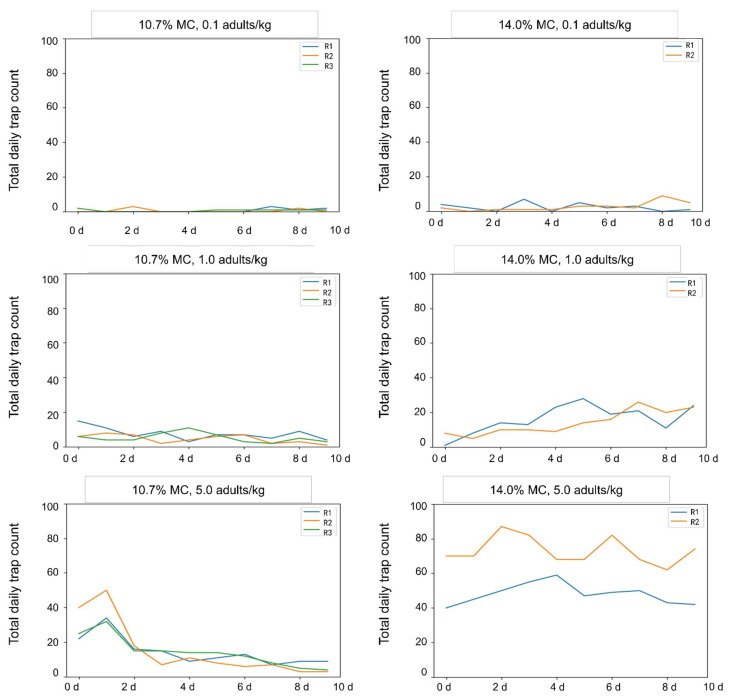
Total daily trap counts during 10 days of detection in paddy bulks with different moisture contents (MCs) at insect densities of 0.1, 1.0 and 5.0 adults/kg.

The trend of total daily trap counts over time varied significantly when the grain moisture varied and the insect density remained the same (Figure 4). The total daily trap counts followed a decreasing trend over time when the insect density was 1.0 and 5.0 adults/kg in paddy with 10.7% MC. This is because the optimum ambient humidity for *C. pusillus* adults is more than 60% [21]. During the experiment, the humidity inside the 10.7% paddy bulks was approximately 40% on average (Table 2), and the activity of *C. pusillus* adults decreased significantly. In the paddy with 14.0% MC, the total daily trap count fluctuated with time. The trap count was significantly higher than those in paddy with 10.7% MC at the same insect density (paired *t*-test, *t* = −3.124, −2.823, and −7.081, and *p* = 0.006, 0.01, and <0.001, for 0.1, 1.0, and 5.0 adults/kg, respectively). As shown in Table 2, in the paddy with 14.0% MC, relative humidity rose to more than 60%, insect activity increased, and their random movement increased, leading to higher trap counts.

Several factors may explain these findings: First, the activity of *C. pusillus* was significantly affected by grain moisture content. Second, temporal and seasonal differences influenced the results. The experiment using paddy with 14.0% MC was conducted in spring, during which grain temperatures were higher than that of the treatment of paddy with 10.7% MC, which was initiated in late autumn. Natural seasonal variations in insect activity likely contributed to the observed differences. As shown in Figure 2, total daily trap counts in the treatment of paddy with 14.0% MC exhibited a fluctuating upward trend, whereas the treatment of grain with 10.7% MC showed a fluctuating decline. Third, a slight fungal infestation (observed as micro-fluff) was detected in the paddy with 14.0% MC during manual sampling. This suggests a potential stimulatory effect of fungal presence on insect activity, as reflected by increased trap counts in this treatment.

### 3.3. Spatial Distribution of Adults Characterized by Trap Count

**Figure 5:**
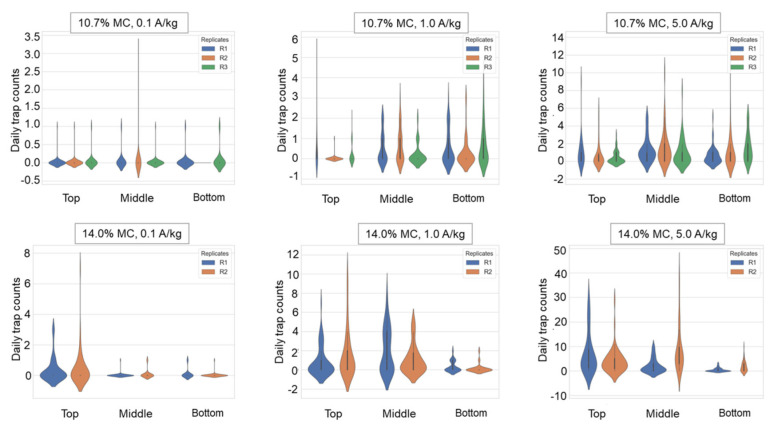
Violin plots of daily trap count from traps deployed in the top, middle, and bottom layers under different introduced insect densities (A/kg, adults/kg) and paddy moisture content (MC) levels.

**Figure 6:**
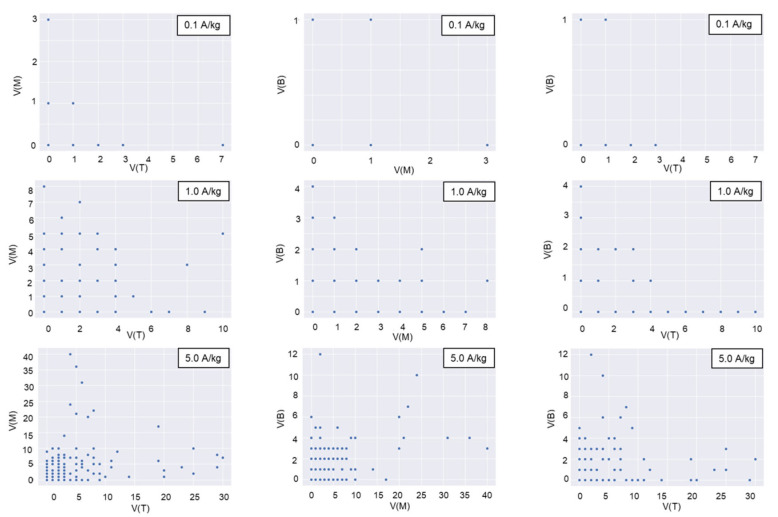
H-scatterplots for 60 cm separation distance between the top and middle layers (left graphs), 60 cm separation distance between the middle and bottom layers (middle graphs), and 120 cm separation distance between the top and bottom layers (right graphs) under different introduced insect densities (A/kg, adults/kg).

When the insect density was low (0.1 and 1.0 adults/kg), beetles were captured more frequently in the middle or bottom layers in the paddy with 10.7% MC. In the paddy with 14.0% MC, beetles were detected more often in the middle and top layers (Table 3). Notably, when the insect density increased to 1 beetle/kg, more than half of the beetles were captured in the middle layer, and the remaining 41.6% were captured in the top layer. When the insect density increased to more than 5 adults/kg, the percentage of beetles captured in the top layer increased in paddy with 10.7% MC, but the percentage of detection in the middle layer was still the highest; in the paddy with 14.0% MC, the percentage of detection in the top layer was the highest. In summary, insects were more likely to be detected in the middle layer under the conditions of a paddy with low moisture content, whereas higher paddy MC conditions led to increased detection in the top layer (Figure 5). This spatial distribution pattern was corroborated by h-scatterplot analysis (Figure 6). Additionally, comparative trap count data (Table 1) revealed significantly higher daily trap counts in replicates using paddy with 14.0% MC at an insect density of 5.0 adults/kg, surpassing counts for all other MC levels tested. The central trap in the middle layer consistently recorded high captures, suggesting the potential aggregation behavior of *C. pusillus* adults at this location.

**Figure 7:**
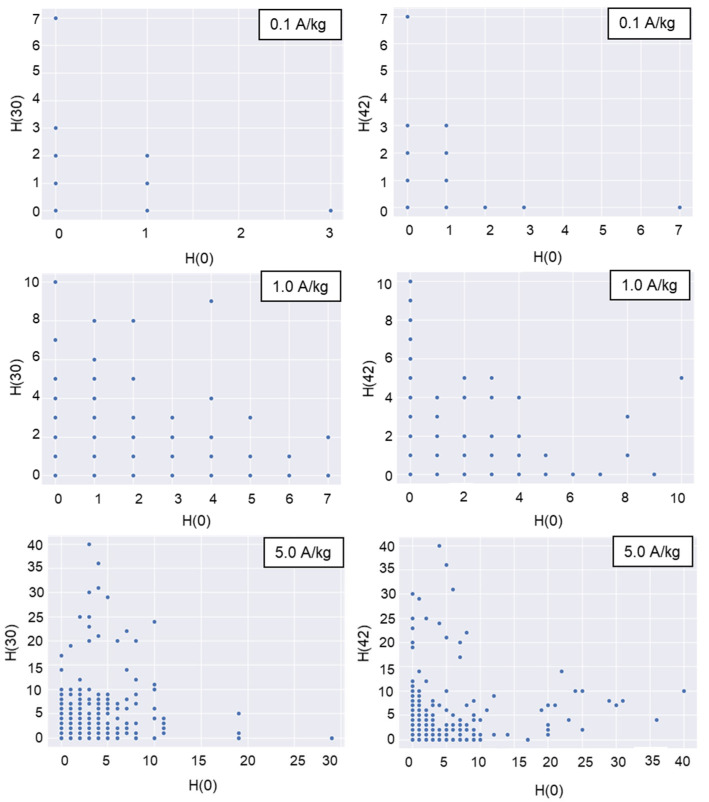
H-scatterplots for 30 cm (left graphs) and 42 cm (right graphs) separation distances in the horizontal direction under different introduced insect densities (A/kg, adults/kg).

When insect density was 0.1 adults/kg, the introduced beetles in the horizontal direction preferred the half-radius locations (Figure 7). This trend was identified according to the increased density of the introduced insects. The rapid dispersal of beetles at higher insect densities at the start of their introduction may have been responsible for this increase, or it may be influenced by the emission of sexual or aggregation semiochemicals [22,23].

As shown in Table 4, trap counts between different locations exhibited weak correlations. Positive Pearson correlation coefficients indicate positively correlated spatial distributions of trap counts, while negative values reflect inverse relationships. For each moisture content level, six sets of correlation coefficients were analyzed across horizontal and vertical directions at all insect densities. Results indicate no significant variation in correlation coefficients with changing MC in either direction (paired *t*-test; horizontal: *t* = 1.032, *p* = 0.331; vertical: *t* = 0.090, *p* = 0.931). In the horizontal direction at identical insect densities, correlation coefficients between center and half-radius locations showed no significant difference from those between two half-radii locations, except at 1.0 adults/kg (*t*-test; 0.1 adults/kg: *t* = −1.069, *p* = 0.397; 1.0 adults/kg: *t* = −7.999, *p* = 0.015; 5.0 adults/kg: *t* = −0.328, *p* = 0.772). Similarly, no significant differences were observed in the vertical direction (*t*-test; 0.1 adults/kg: *t* = −0.832, *p* = 0.493; 1.0 adults/kg: *t* = 5.093, *p* = 0.303; 5.0 adults/kg: *t* = 0.940, *p* = 0.447). Notably, trap counts at half-radius locations were uncorrelated in both directions.

### 3.4. Influence of Storage Conditions on Trap Counts

Table 4 shows the statistical results of daily average trap counts, daily average grain temperature, and humidity under different storage conditions.

**Figure 8:**
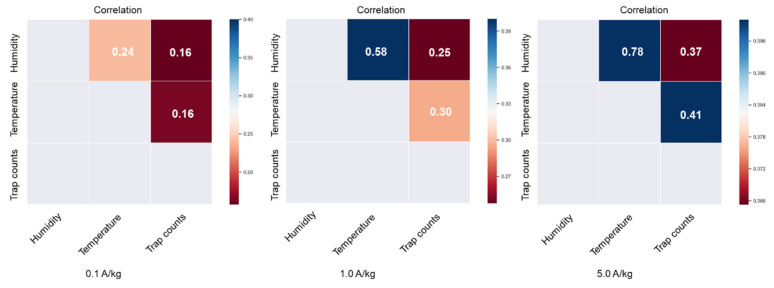
Heat maps of Pearson correlation coefficients between daily trap counts, grain temperature, and humidity obtained from each trap under different insect densities (A/kg, adults/kg).

Table 5 shows the results of the four-way ANOVA. Except for the insect density, the MC, grain temperature, and humidity at the trap location all had a significant influence on trap counts. Although trap counts were different at different insect densities, trap counts were higher at locations with higher grain temperature and higher humidity than at other locations with the same insect density. The increase in trap counts may have occurred because the activity of *C. pusillus* adults was influenced primarily by grain temperature and grain MC [24]. Because adults prefer warmer and moister locations [25], there was a higher probability of obtaining higher trap counts in warmer and moister locations. Trap counts exhibited significant positive correlations with both in situ grain temperature and humidity at the corresponding trap location (Figure 8). Critically, temperature demonstrated the strongest correlation coefficients (0.16, 0.30, and 0.41 in Figure 8), indicating that ambient thermal conditions strongly influence insect activity patterns.

### 3.5. Aggregation Distribution Characterized by Trap Counts

Table 6 provides the parametric regression results (mean values) of Lloyd’s index of mean crowding and Taylor’s power law calculated for different MC levels and different insect densities. In most cases, adults in the paddy bulks were aggregated when the daily trap counts of a single trap were used as the sampling result of insects in most cases, which corresponded to the spatially nonuniform distribution of the trap counts (Figure 6 and Figure 7). As a detritivorous species, *C. pusillus* aggregates in microhabitats rich in organic debris or fungal-contaminated grains, which serve as nutritional hotspots. These impurities (e.g., fragmented kernels, microbial biomass) create discontinuous resource gradients, leading to clustered insect distributions. In addition, aggregation pheromones and sex pheromones [23,26] also influence the insect’s distribution pattern. These factors collectively contribute to the observed heterogeneous distribution.

### 3.6. Relationship Between Insect Densities and Trap Counts

Table 7 presents the average Pearson correlation coefficients between the introduced insect density and the trap count frequencies at each location over trapping periods of 3, 5, 7, and 10 days. The results indicate that the correlation coefficients were generally highest when the trapping period was 10 days. It is important to note that trap interference may have occurred due to attractive or repellent responses resulting from chemical communication among the insects. Future research could further investigate the behavioral mechanisms underlying such interference, which may help refine trap deployment strategies in stored-grain environments.

The frequencies of trap counts (TFs) were different in different trapping periods, and the TF values at different locations were different when we used the total trap counts in the past 10 days to calculate the TF. When the insect densities were 0.1, 1.0, and 5.0 adults/kg, the TF values were 0.09 ± 0.02, 0.64 ± 0.10, and 2.20 ± 0.43, respectively.

The Pearson correlation coefficient values ranged from 0.06 to 0.99, with an average of 0.68. As shown in Table 8, when separately processing data with different MC levels, 27 of 30 Pearson correlation coefficients were greater than 0.5. Regarding the pooled data, however, the Pearson correlation coefficients were much lower. Because trap counts were significantly influenced by the grain MC and the measured grain temperature and humidity at the same location, the regressed correlation decreased when using pooled data.

### 3.7. Discussion

Trap count distribution was characterized from the aspect of both temporal and spatial directions. In the treatment with 14.0% MC, counts fluctuated upwards, while the 10.7% MC treatment showed a declining trend, likely due to seasonal variations, grain moisture content, and fungal infestation. Spatially, at an insect density of 0.1 adults/kg, introduced beetles preferred half-radius locations, with increased density correlating to rapid dispersal or the influence of aggregation and sex pheromones. The variations in trap counts were also affected by grain temperature and moisture content, with higher counts observed in warmer, moister areas preferred by *C. pusillus*. As a detritivore, *C. pusillus* aggregates in microhabitats with organic debris or fungal-contaminated grains, which create discontinuous resource gradients and lead to clustered distributions. The results of this study provide some guidance for insect occurrence assessment based on trap counts in bulk grain. However, the following issues should be addressed for the practical assessment of pest insect occurrence in stored grain based on trap counts: (1) as trap counts are variable during the whole detection period, it is inappropriate to infer distribution dynamics from only the frequency of trap counts over a specific period; (2) we have to combine grain temperature and MC to assess insect distribution dynamics; and (3) it was challenging to accurately assess insect distribution dynamics based on trap counts collected by one electronic probe trap because of the aggregate distribution of insects, and thus more traps at different locations are required. In addition, more species of pests and mites interact with each other [26]; thus, future research should incorporate multiple insect pest species to enhance practical applications in pest control.

## 4. Conclusions

This research was undertaken in order to characterize the spatial and temporal distribution of *C. pusillus* adults in paddy bulks under different storage conditions based on trap counts under the laboratory environment. Electronic probe traps detected *C. pusillus* adults in paddy more sensitively, but the daily trap counts were not stable and varied significantly with grain MC. The distribution of *C. pusillus* adults in paddy was uneven in both horizontal and vertical directions. Except for insect density, MC, grain temperature, and humidity at the trap location significantly affected the trap counts. Distribution patterns of insects in bulk grain were characterized using trap counts. In most cases, insects aggregated in paddy bulks. The average daily trap counts in a 10-day trapping period for each trap had the highest correlation with insect density, and the correlation was different among different trap locations.

However, these findings derive from a simplified single-species system. In practical storage ecosystems, coexisting insects and mites may interact through competition or predation, thereby altering pest behavior, spatial distribution, and trap efficacy. Therefore, future studies should incorporate multiple pest species and quantify their interspecific dynamics.

## Figures and Tables

**Table 1:** Detection results using different detection methods.

Insect Density (Adults/kg)	MC (%)	Replicate	Electronic Probe Trapping		Manual Sampling	
			Number of Traps That Detected Beetles per Day	Total Daily Trap Count	Number of Successful Detections	Number of Detected Beetles
0.1	10.7	R1	0.6	0.6	0	0
		R2	0.3	0.5	0	0
		R3	0.7	0.7	0	0
	14.0	R1	1.7	2.4	0	0
		R2	1.7	2.7	0	0
1.0	10.7	R1	4.8	7.6	0	0
		R2	3.2	4.6	0	0
		R3	3.8	5.3	0	0
	14.0	R1	5.7	16.2	2	2
		R2	5.5	14.1	3	3
5.0	10.7	R1	8.0	14.5	5	9
		R2	6.7	15.3	4	8
		R3	7.2	14.4	9	18
	14.0	R1	8.4	48.8	8	16
		R2	11.7	73.1	10	33

**Table 2:** Daily average trap count, daily average grain temperature, and humidity (means ± SE).

Insect Density (Adults/kg)	MC (%)	Trap Count ^a^	Temperature (°C)	Humidity (%)
0.1	10.7	0.0 ± 0.23	25.9 ± 1.51	43.2 ± 2.07
	14.0	0.2 ± 0.62	26.4 ± 3.05	62.1 ± 4.01
1.0	10.7	0.4 ± 0.75	26.3 ± 1.28	43.4 ± 2.95
	14.0	1.0 ± 1.77	28.0 ± 2.31	61.7 ± 3.45
5.0	10.7	1.0 ± 1.55	26.0 ± 1.75	42.3 ± 4.18
	14.0	4.0 ± 6.32	28.9 ± 2.24	61.7 ± 3.14

^a^ The mean of daily trap count per trap.

**Table 3:** Capture percentage (mean ± SE, %) of *C. pusillus* adults at different layers (CP_L_) under different storage conditions.

Insect Density (Adults/kg)	MC (%)	Top Layer	Middle Layer	Bottom Layer
0.1	10.7	21.7 ± 3.5	48.1 ± 19.0	30.2 ± 16.6
	14.0	78.2 ± 3.2	9.5 ± 5.3	12.3 ± 8.6
1.0	10.7	18.0 ± 8.9	40.3 ± 14.0	41.7 ± 9.4
	14.0	41.6 ± 15.1	50.6 ± 12.3	7.7 ± 2.8
5.0	10.7	22.9 ± 6.6	41.3 ± 4.5	35.8 ± 9.6
	14.0	51.5 ± 22.3	40.2 ± 19.1	9.3 ± 3.1

**Table 4:** Pearson correlation coefficient of daily trap counts between two locations in the horizontal and vertical directions.

MC (%)	Horizontal Direction (H) ^a^						Vertical Direction (V) ^b^					
	0.1 Adults/kg		1.0 Adults/kg		5.0 Adults/kg		0.1 Adults/kg		1.0 Adults/kg		5.0 Adults/kg	
	[H(0), H(30)]	[H(0), H(42)]	[H(0), H(30)]	[H(0), H(42)]	[H(0), H(30)]	[H(0), H(42)]	[V(0), V(60)]	[V(0), V(120)]	[V(0), V(60)]	[V(0), V(120)]	[V(0), V(60)]	[V(0), V(120)]
10.7	−0.03	0.12 *	−0.05	0.02	0.22 **	0.23 **	0.02	−0.03	0.03	−0.06	0.25 **	0.21 **
14.0	−0.00	−0.02	−0.07	0.02	0.02	0.09	−0.04	0.24 *	0.07	−0.05	0.17 *	−0.01

* Similar between trap counts at two locations at α ≤ 0.05. ** Similar between trap counts at two locations at α ≤ 0.01. ^a^ H represents the horizontal direction. The first bracket denotes the number of the start location H(0), and the second bracket specifies the distance between the start trap and the second trap location in the indicated direction, H(30) and H(42). ^b^ V represents the vertical direction. The first bracket denotes the number of the start location V(0), and the second bracket specifies the distance between the start trap and the second trap location in the indicated direction, V(60) and V(120).

**Table 5:** Statistical result of the four-way ANOVA to determine whether the storage condition influences the trap counts of *C. pusillus*.

Factors	DF ^a^	F ^b^	P ^c^
Insect density	2	143.813	<0.0001
Moisture content	1	138.611	<0.0001
Temperature	3	59.515	<0.0001
Humidity	3	6.556	0.0002
Error	2240	NaN	NaN

^a^ The degree of freedom. ^b^ The F value of the four-way ANOVA. ^c^ The *p* value of the four-way ANOVA.

**Table 6:** Characterized patterns of aggregation distribution using different indices.

Insect Density (Adults/kg)	MC (%)	Lloyd’s Index of Mean Crowding				Taylor’s Power Law		
		b_0_	Pattern	b_1_	Pattern	lna	b	Pattern
0.1	10.7	0.07	Aggregation	−0.07	Uniform	−0.29	0.89	Uniform
	14.0	−0.36	Repulsion	6.34	Aggregation	1.49	1.55	Aggregation
1.0	10.7	−0.06	Repulsion	2.99	Aggregation	0.73	1.26	Aggregation
	14.0	64.78	Aggregation	−38.50	Uniform	8.73	−15.57	Uniform
5.0	10.7	−0.22	Repulsion	1.20	Aggregation	−0.08	1.08	Aggregation
	14.0	−2.51	Repulsion	3.67	Aggregation	0.57	2.18	Aggregation

**Table 7:** Pearson correlation coefficients associated with the introduced insect density and the frequency of trap counts on days 3, 5, 7, and 10.

MC (%)	Period Length			
	3 Days	5 Days	7 Days	10 Days
10.7	0.29 **	0.42 **	0.48 **	0.66 **
14.0	0.41 **	0.43 **	0.42 **	0.46 **
Pooled	0.27 **	0.30 **	0.30 **	0.37 **

** Similar between insect density and trapping frequency at α ≤ 0.01.

**Table 8:** Pearson correlation coefficient between introduced densities of *C. pusillus* and trapping frequency at different locations.

MC (%)	Depth	Location				
		1	2	3	4	5
10.7	Top	0.77 *	0.33	0.89 **	0.81 **	0.06
	Middle	0.86 **	0.91 **	0.88 **	0.77 *	0.73 *
	Bottom	0.82 **	0.50	0.63	0.96 **	0.58
14.0	Top	0.10	0.93 **	0.63	0.95 **	0.99 **
	Middle	0.60	0.47	0.89 *	0.79	0.62
	Bottom	0.50	0.70	0.89 *	0.90 *	0.67
Pooled	Top	0.32	0.51 *	0.42	0.59 *	0.49
	Middle	0.62 *	0.59 *	0.60 *	0.53 *	0.41
	Bottom	0.68 **	0.57 *	0.74 **	0.89 **	0.58 *

* Similar between insect density and trapping frequency at α ≤ 0.05. ** Similar between insect density and trapping frequency at α ≤ 0.01.

## Data Availability

The original contributions presented in this study are included in the article. Further inquiries can be directed to the corresponding author.
